# Characterizing alpha helical properties of Ebola viral proteins as potential targets for inhibition of alpha-helix mediated protein-protein interactions

**DOI:** 10.12688/f1000research.5573.3

**Published:** 2015-01-27

**Authors:** Sandeep Chakraborty, Basuthkar J. Rao, Bjarni Asgeirsson, Abhaya Dandekar

**Affiliations:** 1 Plant Sciences Department, University of California, Davis, 95616, USA; 2 Department of Biological Sciences, Tata Institute of Fundamental Research, Homi Bhabha Road, Mumbai, 400 005, India; 3 Science Institute, Department of Biochemistry, University of Iceland, Dunhaga 3, IS-107 Reykjavik, Iceland

## Abstract

Ebola, considered till recently as a rare and endemic disease, has dramatically transformed into a potentially global humanitarian crisis. The genome of Ebola, a member of the Filoviridae family, encodes seven proteins. Based on the recently implemented software (PAGAL) for analyzing the hydrophobicity and amphipathicity properties of alpha helices (AH) in proteins, we characterize the helices in the Ebola proteome. We demonstrate that AHs with characteristically unique features are involved in critical interactions with the host proteins. For example, the Ebola virus membrane fusion subunit, GP2, from the envelope glycoprotein ectodomain has an AH with a large hydrophobic moment. The neutralizing antibody (KZ52) derived from a human survivor of the 1995 Kikwit outbreak recognizes a protein epitope on this AH, emphasizing the critical nature of this secondary structure in the virulence of the Ebola virus. Our method ensures a comprehensive list of such `hotspots'. These helices probably are or can be the target of molecules designed to inhibit AH mediated protein-protein interactions. Further, by comparing the AHs in proteins of the related Marburg viruses, we are able to elicit subtle changes in the proteins that might render them ineffective to previously successful drugs. Such differences are difficult to identify by a simple sequence or structural alignment. Thus, analyzing AHs in the small Ebola proteome can aid rational design aimed at countering the `largest Ebola epidemic, affecting multiple countries in West Africa' ( http://www.cdc.gov/vhf/ebola/outbreaks/2014-west-africa/index.html).

## Introduction

The Ebola virus was first discovered in 1976 ^1^, and has been since known as a rare, but deadly disease ^2^. However, the current outbreak in West African countries (Guinea, Liberia, Nigeria, Sierra Leone and Senegal) has rapidly deteriorated into a full blown epidemic ^3^, and poses grave humanitarian dangers to these countries ^4^. Ebola, along with the Marburg virus, belongs to the *Filoviridae* family ^5^, and causes haemorrhagic fever ^2^ by quickly suppressing innate antiviral immune responses to facilitate uncontrolled viral replication ^6^.

Interestingly, the genome of the Ebola virus encodes seven proteins ^7^, although their extreme ‘plasticity allows multiple functions’ ^8, 9^. Protein structures are formed by well ordered local segments, of which the most prevalent are alpha helices (AH) and *β* sheets. AHs are right-handed spiral conformations which have a hydrogen bond between the carbonyl oxygen (C=O) of each residue and the alpha-amino nitrogen (N-H) of the fourth residue away from the N-terminal. AH domains are often the target of peptides designed to inhibit viral infections ^10– 12^. Recently, we have provided open access to software that has reproduced previously described computational methods ^13^ to compute the hydrophobic moment of AHs (PAGAL ^14^).

In the current work, we characterize the helices in the Ebola proteome using PAGAL, and demonstrate that the helices with characteristically unique feature values are involved in critical interactions with the host proteins. The PDB database is queried for the keyword ‘Ebola’, and the structures obtained are analyzed using DSSP ^15^ for identifying AHs. We process all PDB structures, and do not filter out redundant structures based on sequence. These helices are analyzed using PAGAL, and the results are sorted based on three criteria - hydrophobic moment and high proportion of positive or negative residues. The helices that are ranked highest in these sorting criteria are involved in critical interactions with either antibodies or host proteins. For example, the Ebola virus membrane fusion subunit, GP2, from the envelope glycoprotein ectodomain has an AH with the largest hydrophobic moment in all helices analyzed ^16^. This helix has part of the epitope recognized by the neutralizing antibody (KZ52) derived from a human survivor of the 1995 Kikwit outbreak, emphasizing the critical nature of this helix in the virulence of Ebola ^17^. Another example, obtained by choosing the helix with the highest proportion of negatively charged residues, is the interaction between the human karyopherin alpha nuclear transporters C terminus and the Ebola virus VP24 protein (eVP24) ^18^, which suppresses tyrosine-phosphorylated STAT1 nuclear import ^19^. These helices probably are, or can be, the target of molecules designed to inhibit AH mediated protein-protein interactions ^20^. Our method provides a comprehensive list of such targets. Further, each protein can be individually queried using PAGAL, and thus identify helices that might have a poor global rank, but still be critical in the particular proteins context.

Although, Ebola and Marburg viruses are members of the *Filoviridae* family ^21^, they have different antigenicity of the virion glycoprotein ^22^. By comparing the AHs in proteins of Marburg and Ebola viruses, we are able to elicit subtle changes in the proteins that might render them ineffective against previously successful drugs. These differences are not apparent from a simple sequence or structural alignment. Thus, in the current work, we elucidate a simple methodology that can aid rational design of drugs and vaccine, an important aspect of the global effort to counter the deadly Ebola epidemic.

## Materials and methods

We searched for the keyword ‘Ebola’ in the PDB database ( Table 1). Subsequently, each protein was split based on the chain id, resulting in 146 single chained proteins (See ALPHA.zip in Dataset 1). We have not reduced the set based on sequence similarity since the proteins might have different conformations based on their ligands. Note, this list might include non-Ebola proteins which might have been co-crystallized with the Ebola protein. However, they have been put through the same analysis since they might provide insights into the Ebola proteins themselves.

**Table 1.** PDB ID of Ebola proteins analyzed.

PDB ID	Description
1EBO,2EBO,3VE0,3CSY.. 2I8B,3V7O 3FKE,3L25,4LG2,4IBK... 3VNE,4D9O,4M0Q,4U2X.. 4QAZ,4QAZ 1ES6,1H2D,3TCQ,4LDM...	Ebola virus envelope protein Minor nucleoprotein VP30 Polymerase cofactor VP35 Membrane-associated protein VP24 Nucleoprotein Matrix protein VP40

These proteins were then analyzed using DSSP ^15^, and resulted in 758 helices in all (See ALPHA.zip in Dataset 1). These helices were then analyzed using PAGAL. The PAGAL algorithm has been detailed previously ^14^. Briefly, the Edmundson wheel is computed by considering a wheel with centre (0,0), radius 5, first residue coordinate (0,5) and advancing each subsequent residue by 100 degrees on the circle, as 3.6 turns of the helix makes one full circle. We compute the hydrophobic moment by connecting the center to the coordinate of the residue and give it a magnitude obtained from the hydrophobic scale (in our case, this scale is obtained from ^13^). These vectors are then added to obtain the final hydrophobic moment.

The color coding is as follows: all hydrophobic residues are colored red, while hydrophilic residues are colored in blue: dark blue for positively charged residues, medium blue for negatively charged residues and light blue for amides.

The raw file generated by analyzing all 146 proteins through PAGAL is provided as PAGALRAWDATA.txt ( Dataset 1), and contains the hydrophobic moment, percent of positive charges and the total number of charged residues for every helix. These are then sorted based on the charge (negative or positive) or the hydrophobic moment. We ignore the helices that have none or a single charged residue, and those that are smaller than 10 residues in length. The proportion of charged residues is computed based on the total number of charged residues, and not the length of the helix.

All protein structures were rendered by PyMol ( http://www.pymol.org/). The sequence alignment was done using ClustalW ^23^. The alignment images were generated using Seaview ^24^. Protein structures have been superimposed using MUSTANG ^25^.

## Results and discussion

PAGAL analysis of Ebola-related alpha helices A PDB database search using the keyword ‘Ebola’ generate 146 single chained proteins, which were analyzed using Define Secondary Structure of Proteins, resulting in 758 alpha helices (ALPHA.zip). Note, this list might include non-Ebola proteins which might have been co-crystallized with the Ebola protein. These helices were analyzed using PAGAL (PAGALRAWDATA.txt), which details the hydrophobic moment, percent of positive charges and the total number of charged residues for every helix.

### Helices with large hydrophobic moment

**Figure 1.**
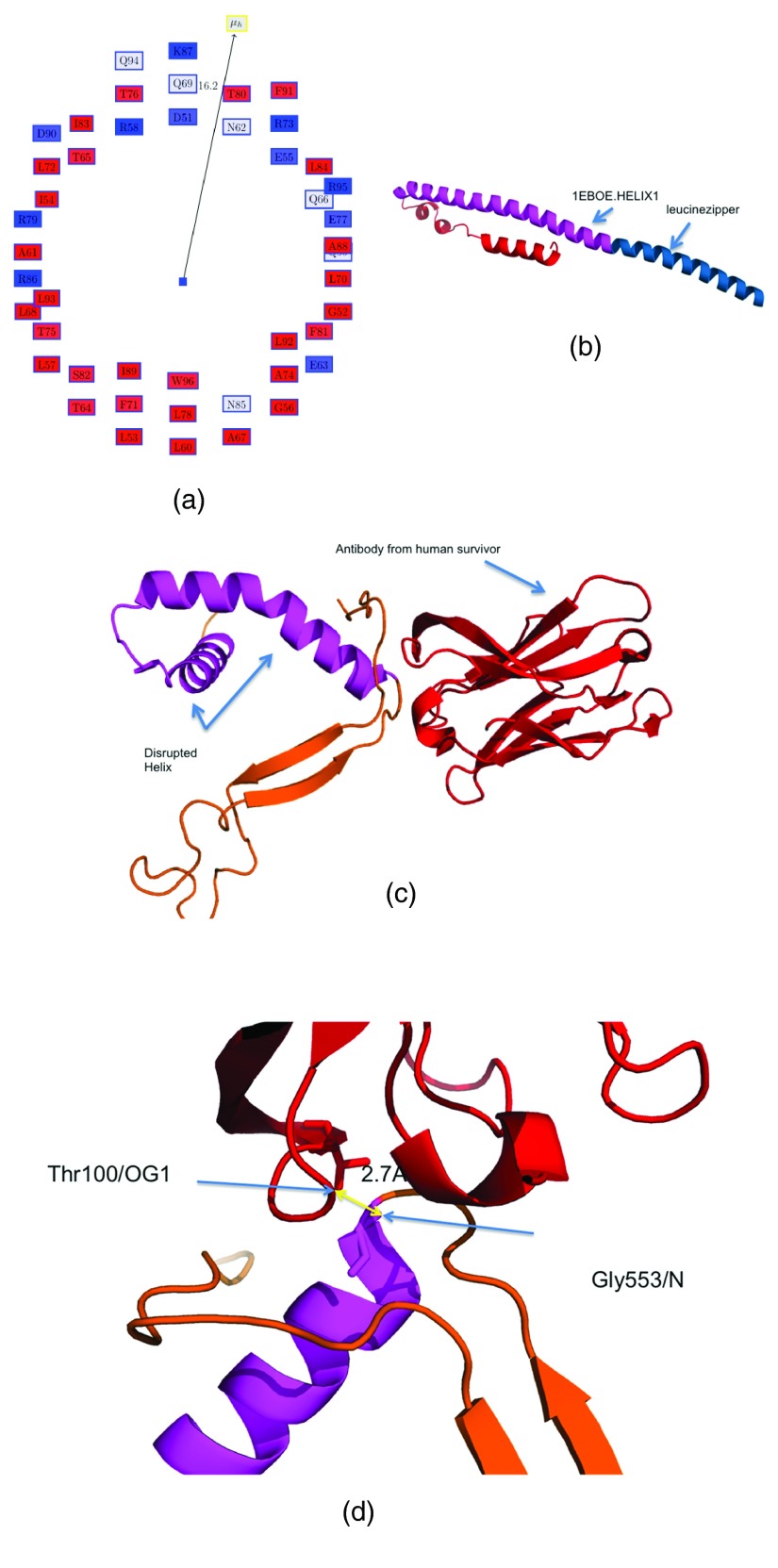
Helix with large hydrophobic moment in GP2 from the Ebola virus membrane fusion glycoprotein. ( **a**) Edmundson wheel for 1EBOE.HELIX1. The hydrophobic moment vector is not to scale. The color coding is as follows: all hydrophobic residues are colored red, while hydrophilic residues are colored in blue: dark blue for positively charged residues, medium blue for negatively charged residues and light blue for amides. ( **b**) Structure of PDBid:1EBOE, 1EBOE.HELIX1 is marked in magenta and the leucine zipper is in blue. ( **c**) 1EBOE.HELIX1 is disrupted by an antibody derived from a human survivor of the 1995 Kikwit outbreak (PDBid:3CSY). ( **d**) Gly553/N on 1EBOE.HELIX1 makes a possible hydrogen bond to Thr100/OG1 at a distance of 2.7 Å.

We began by analyzing the helices which have a large hydrophobic moment (hydrophobic scale is obtained from ^13^) ( Table 2). The Edmundson wheel for the helix 1EBOE.HELIX1 from the structure of GP2 from the Ebola virus membrane fusion glycoprotein (PDBid:1EBO) ^16^ is shown in Figure 1a. Figure 1b shows the residues comprising these helices (in magenta) in the apo form (PDBid:1EBO) ^16^. The neutralizing antibody (KZ52) derived from a human survivor of the 1995 Kikwit outbreak (PDBid:3CSY) ^17^ recognizes an epitope on this AH, emphasizing the critical nature of this AH in the virulence of the Ebola virus ( Figure 1c,d). The antibody most likely inhibits the rearrangement of GP2 segments, which abrogates the fusion of the internal loop in the host membrane ^17^. Table 3 shows the residues in the specified helix (residues 553-597, chain J, PDBid:3CSY) making possible hydrogen bonds with different residues in the human Fab KZ52 heavy chain (residues 1-228, chain A, PDBid:3CSY). Among all the interactions, only Gly553 is on 1EBOE.HELIX1 (at a distance of 2.7 Å from Thr100/OG1), although the others are sequentially proximal. These few interactions are sufficient to bind to this helix, rendering the virus non-virulent, and leading to human recovery. The importance of interfacial hydrophobicity in viral proteins involved in host entry through membrane fusion has recently been discussed in detail, and remains ‘an underutilized therapeutic target’ ^26^. 1EBOE.HELIX0 ( Table 2) also has a high hydrophobic moment, but is actually an isoleucine zipper derived from GCN4 ^27^ ( Figure 1b).

**Table 2.** Identifying helices with unique properties. Property based on which the sorting is done is either the Hydrophobic moment (HM) and the percentage of negative (NEG) or positive residues (POS). HM: Hydrophobic moment, RPNR: Ratio of the positive to the negative residues, Len: length of the helix, NCH: number of charged residues. GP: glycoprotein from Ebola, VP24: Membrane-associated protein from Ebola, VP35: Polymerase cofactor.

Property	Protein	Helix	Len	HM	RPNR	NCH
HM	GP GP	1EBOE.HELIX1 1EBOE.HELIX0	46 29	16.2 11.5	0.5 0.5	11 13
NEG	VP24 VP35	4U2XA.HELIX5 3FKEA.HELIX2	16 14	4.4 3.2	0 0.2	2 4
POS	VP24 VP35	4U2XA.HELIX7 3FKEA.HELIX1	19 15	6.5 7.8	0.8 0.8	5 4

**Table 3.** Interactions obtained from the crystal structure of the Ebola virus glycoprotein in complex with a neutralizing antibody from a human survivor. The helix with a large hydrophobic moment, as determined from PDBid:1EBOE, is disrupted in the structure from PDBid:3CSY through possible hydrogen bonds with different residues in the human Fab KZ52 heavy chain (antibody, chain A). The helix residues are: 553-597 in chain J, PDBid:3CSY.

AtomEbola	AtomAntibody	Dist (Å)
ASP/552/OD1 GLY/553/N ASN/550/O ASP/552/OD1 ASN/550/ND2 ASN/550/ND2 ASP/552/OD2	SER/53/OG THR/100/OG1 ASN/31/O SER/53/CB PRO/97/O ASN/31/O SER/53/OG	2.5 2.7 2.9 2.9 3.0 3.2 3.2

### Helices with high proportion of negatively charged residues. Identifying difference among related species

**Figure 2.**
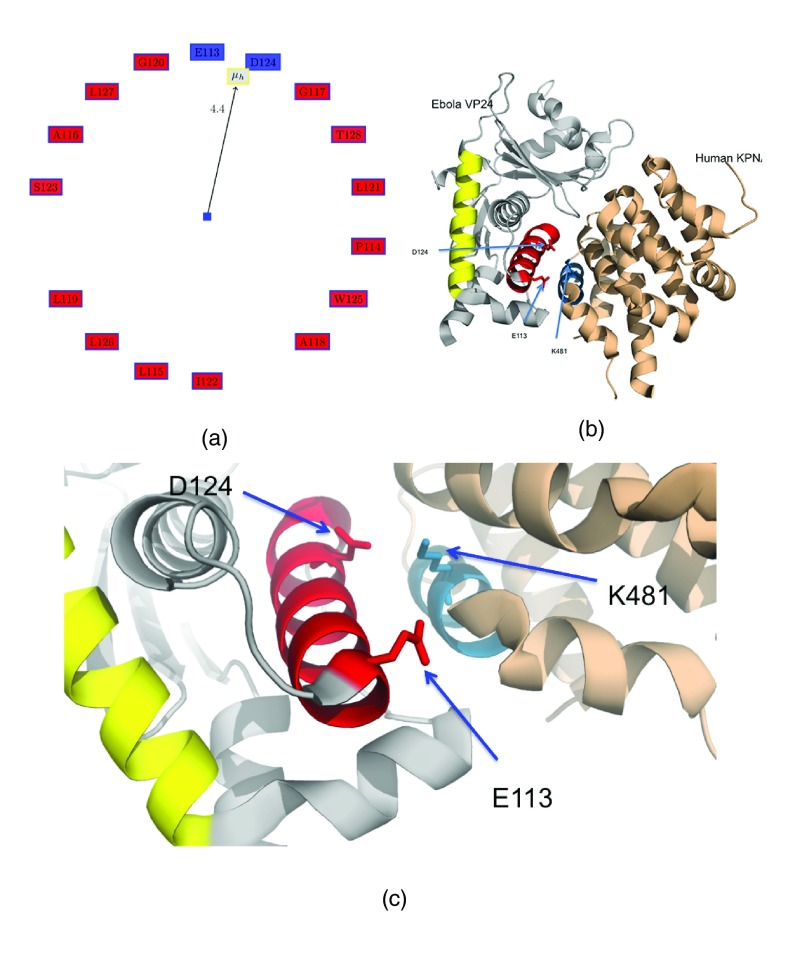
Helix 4U2XA.HELIX5 from membrane-associated protein VP24 with a high proportion of acidic residues. ( **a**) Edmundson wheel for 4U2XA.HELIX5. ( **b**) Complex of VP24 (PDBid:4U2XA) and human karyopherin alpha nuclear transporters (KPNA) C terminus (PDBid:4U2XD). D124 from VP24 probably has an electrostatic interaction with K481 from KPNA. This interaction is sufficient to interfere with the immune response to Ebola infection.

We then analyzed the helices having a high proportion of negatively charged residues, sorted based on the length of the helix when the percentage of negatively residues are the same ( Table 2). Figure 2a shows the Edmundson wheel for the helix 4U2XA.HELIX5 (which has only two charged residues - the basic E113 and D124), while Figure 2b,c shows this helix in the protein complex marked in magenta. Note, that we exclude AHs with either zero or one charged residues (see Methods). Protein PDBid:4U2XD is the human karyopherin alpha nuclear transporter (KPNA) C terminus in complex with the Ebola virus VP24 protein (eVP24) ^18^. eVP24 interferes with the immune response by selectively targeting tyrosine-phosphorylated STAT1 nuclear import ^19^. It does not hinder the transport of other cargo that may be required for viral replication. 4U2XA.HELIX5 is responsible for forming the complex with the KPNA protein through a helix (4U2XD.HELIX9, in blue), and K481 from KPNA is in contact with D124 from eVP24 (distance between K481/NZ and D124/OD2 is 3.98 Å). Their interaction is probably electrostatic, since the atoms have opposite charges. VP24 has also been shown to directly bind to STAT1, further compromising the immune response ^28^. Recently, KPNA was docked to Reston Ebola VP24 (PDBid:4D9OA) ^28^ using the VP24 from Zaire Ebola (PDBid:4U2XA) ^18^ as a template ^29^. The docked structure showed that a single mutation might be one of the critical factors responsible for the non-pathogenic nature of Reston Ebola in humans ^30, 31^. Also, it was shown that the VP24 from Marburg virus (PDBid:3VNEA) ^28^, which has a different immunosuppressive mechanism than the Ebola virus ^32^, has different properties in the helices responsible for binding KPNA in the Zaire Ebola VP24.

The next helix having a high proportion of negatively charged residues (3FKEA.HELIX2) is from a VP35, a classic example of a moonlighting protein, that can be a component of the viral RNA polymerase complex, a viral assembly factor, or inhibitor of host interferon production ^33^. This helix is part of the dsRNA-binding domain of VP35 that is involved in the formation of the asymmetric VP35 RBD dimeric interface in Reston Ebola virus through a hydrogen-bonding network of residues and a solvent molecule ^34^. Interestingly, this helix is homologous (100.0% similar and 78% identity in 9 amino acid overlap) to helix ‘1A’ of an ATP-dependent transcriptional activator ^35^. This helix interacts with another ‘1B’ helix from a different monomer in an anti-parallel fashion to facilitate dimerization.

**Figure 3.**
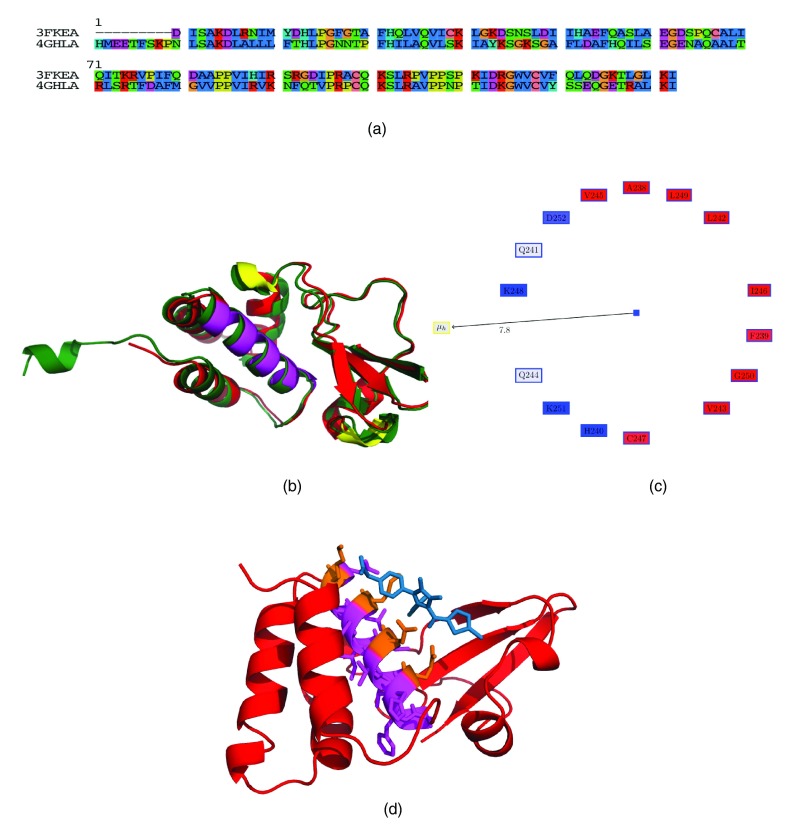
Polymerase cofactor VP35 (PDBid:3FKE). VP35 has several moonlighting functions related to immune evasion. ( **a**) Sequence alignment of VP35 from Marburg (PDBid:4GHLA) and Ebola (PDBid:3FKEA). ( **b**) Structural alignment using MUSTANG. The helices that have differing properties are marked in yellow. 3FKEA.HELIX1 spanning residues 238-252 is marked in magenta. This is a helix with a high proportion of positively charged residues that have been observed to have important interactions in the structure ^33^. ( **c**) Edmundson wheel for 3FKEA.HELIX1. ( **d**) 1D5 (in blue) in complex with VP35 (PDBid:4IBFA).

VP35 consists of several helices, and is reasonably conserved in the Marburg virus from the same *Filoviridae* family (42% identity, 58% similarity) ( Figure 3a). Often, it is difficult to identify the regions of the protein that differ from a sequence or structural alignment ( Figure 3b), in case there is interest in understanding different responses of the proteins to known drugs or even the immune system. Table 4 compares the characteristics of the helices in the VP35 from Ebola and Marburg (the helix numbering is offset by one, due to a small N-terminal helix in the Marburg protein (which might be due to crystallization technique differences and probably is not critical). Thus, we have numbered these helices using alphabets. It can be seen that most of the helices have the same properties, barring helices E and F, where the acidic residue is present in the E helix in Marburg and in the F helix in Ebola. These helices are marked in yellow in Figure 3b. Also, it can be seen that helix C, which has a high proportion of acidic residues in VP35, has a fewer number of those residues in Marburg. The difference in the pathogenicity of these viruses are encoded in the structure of the expressed proteins, and the design of drugs and vaccines to counter virulence should take these differences into account.

**Table 4.** Detecting differences in related proteins based on characteristics of alpha helices. Comparing the VP35 protein from Marburg (PDBid:4GHLA) and Ebola (PDBid:3FKEA). Note the helices are offset by one, due the presence of an extra helix in the Marburg VP35. Thus, we name the helices using alphabets. It can be seen that most helices have the same properties, barring helices E and F, where the acidic residue is present in the E helix in Marburg and in the F helix in Ebola. HM: Hydrophobic moment, RPNR: Ratio of the positive to the negative residues, Len: length of the helix, NCH: number of charged residues.

Helix Name	Real Helix	Len	HM	RPNR	NCH
A	4GHLA.HELIX1 3FKEA.HELIX0	10 9	2.8 5	0.5 0.7	2 3
B	4GHLA.HELIX2 3FKEA.HELIX1	15 15	5.3 7.8	1 0.8	3 4
C	4GHLA.HELIX3 3FKEA.HELIX2	13 14	4.6 3.2	0.5 0.2	2 4
D	4GHLA.HELIX4 3FKEA.HELIX3	11 11	5.1 3.6	1 1	2 2
E	4GHLA.HELIX5 3FKEA.HELIX4	3 3	2.7 1.1	0 -1	1 0
F	4GHLA.HELIX6 3FKEA.HELIX5	3 3	1 1.3	-1 0.5	0 2
G	4GHLA.HELIX7 3FKEA.HELIX6	6 6	4.7 4.7	1 1	2 2
H	4GHLA.HELIX8 3FKEA.HELIX7	3 3	2.8 3.4	0.5 0.5	2 2

### Helices with high proportion of positively charged residues

4U2XA.HELIX7 from VP24 is a helix having a high proportion of positively charged residues ( Table 2), and contains two (L147P and R154L) of three mutations (L147P, M71I and R154L) that sensitizes guinea pigs to the Zaire Ebola virus ^36^. This helix is marked in yellow in Figure 2c. The second helix (3FKEA.HELIX1) is from VP35, which was discussed previously ^33^. This helix spans residues 238-252 and includes Lys248 and Lys251, a basic patch which is ‘100% identical among members of the Ebola viral isolates’ ^33^, and Ala238, Gln241, Leu242, Val245, Ile246, Leu249 which interacts with a *β* sheet to create a hydrophobic subdomain ^33^. This helix is marked in magenta in Figure 3b, and the Edmundson wheel is shown in Figure 3c. Recently, antifiloviral compounds were shown to bind and inhibit the polymerase cofactor activity of VP35 ^37^. Figure 3d shows one of the compounds (1D5) in complex with VP35 (PDBid:4IBFA). It can be seen that atoms in the compounds make hydrogen bonds with residues on the AH spanning residues 238–252 ( Table 5). These structures were used to derive a receptor-ligand pharmacophore, which was found to have similar features to the ligand based pharmacophore derived from four FDA approved drugs that inhibit the Ebola virus ^38^. Once again, we demonstrate that unique values of an AH is a strong indicator of its significance in the viral functionality.

**Table 5.** VP35 (PDBid:4IBFA) in complex with a component (1D5) that inhibits its polymerase cofactor activity. Atoms making hydrophobic (HPhobic) and hydrogen bonds (HBond) with the inhibitor. The residues in the charged side of the Edmundson wheel in Figure 3c makes hydrogen bonds to 1D5.

VP35 atom	1D5 atom	Dist (Å)	Interaction type
LYS/251/NZ GLN/241/NE2 ASP/302/O LYS/251/CD LYS/251/CE GLN/244/OE1 ALA/221/CB GLN/241/OE1 GLN/241/CD GLN/244/NE2	OAD OAB CLA OAD OAD CAM OAA OAB OAB OAB	2.6 2.7 3.0 3.2 3.3 3.3 3.5 3.5 3.5 3.5	HBond HBond HPhobic HPhobic HPhobic HPhobic HPhobic HBond HPhobic HBond

### Multifunctional/moonlighting

The multifunctional roles played by many of these Ebola proteins is probably due to stretches of intrinsically disordered regions within the structure - ‘fuzzy objects with fuzzy structures and fuzzy functions’ ^39^. The conformational plasticity ^9^ and moonlighting abilities of these proteins are key determinants for immune evasion ^40^.

**Figure 4.**
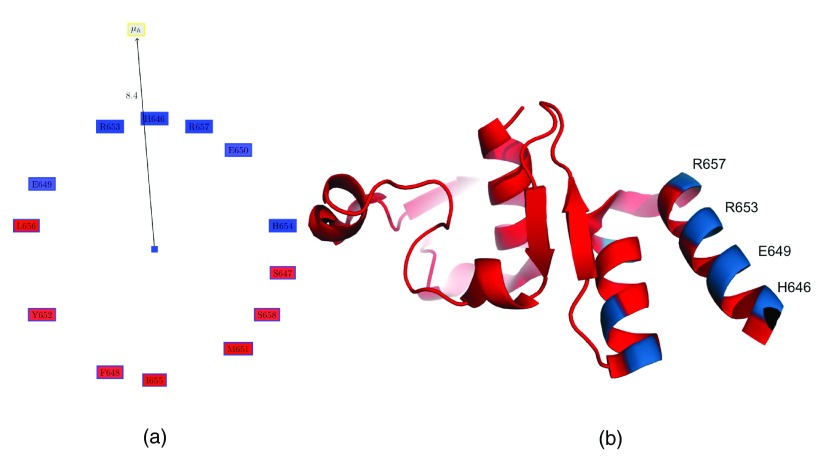
C-terminal domain of the Zaire Ebola virus nucleoprotein ^41^. ( **a**) Edmundson wheel for 4QAZA.HELIX0 (residues 646-658). ( **b**) Protein structure for PDBid:4QAZA.

The above examples have analyzed all helices from the Ebola proteome. However, it also possible to analyze the helices in a single protein, and probe those for unique features. Table 6 shows the values obtained from PAGAL for helices of the C-terminal domain of the Zaire Ebola virus nucleoprotein ^41^. It can be seen that 4QAZA.HELIX0 (residues 646-658) has a reasonably high hydrophobic moment (although it will not rank highly if we analyze all helices present in this proteome), and also a high number of charged residues ( Figure 4a,b). It has been observed that ‘the side chains of Glu645, His646, Glu649, Lys684, Glu695, Glu709, Lys728 and Gln739 are partly disordered so that some or all of their atoms are not visible in the electron density’ ^41^. Glu645, His646, Glu649 are part of this helix, and are thus critical to the disorderedness of the protein, which is critical for its moonlighting roles. Note, that Glu has been observed to be the second most disorder promoting residue (after proline) ^42^. Furthermore, Tyr652 and Leu656, which lie in this helix, are residues that have been hypothesized to be part of the protein-protein interaction site involving this protein ^41^.

**Table 6.** Properties of the helices of the C-terminal domain of the Zaire Ebola virus nucleoprotein (PDBid:4QAZA). 4QAZA.HELIX0 comprising of residues 646-658 has a reasonably large hydrophobic moment, and has been hypothesized to be part of the protein which is involved in protein-protein interactions ^41^. Further, these helices have residues with disordered sidechains ^41^, which are known to be critical for moonlighting functions ^39^. HM: Hydrophobic moment, RPNR: Ratio of the positive to the negative residues, Len: length of the helix, NCH: number of charged residues.

Helix	Len	HM	RPNR	NCH
4QAZA.HELIX0 4QAZA.HELIX1 4QAZA.HELIX2 4QAZA.HELIX3 4QAZA.HELIX4 4QAZA.HELIX5 4QAZA.HELIX6	13 12 3 3 4 3 11	8.4 0.8 2.5 2.4 1.8 0.1 2.1	0.7 0.7 0 0 0.3 -1 1	6 3 1 1 3 0 4

## Conclusions

The ability of a genome as small as the Ebola virus to inflict a dishearteningly high percentage of mortality in human subjects is a humbling experience in the context of the tremendous technological advancements achieved in the last few decades ^3, 4^. The Ebola virus potently suppresses the human immune response ^2, 6, 43^ by binding with key human proteins involved in the immune pathway ^18^. These protein-protein interactions are often mediated through well structured secondary regions within the protein structures (alpha helices), and the design of molecules that inhibit these ‘hotspots’ ^20, 44^ has been a well known strategy to develop drugs to counter bacterial and viral infections ^10– 12^. For example, synthetic peptides derived from the oligomerization domain of polymerase subunits has been shown to inhibit viral proteins ^45, 46^. In addition, there might exist other protein domains that might be exploited by non-native viral peptides to obstruct viral functionality. In the current work, we characterize alpha helices in the Ebola virus proteome using a recently implemented open access software (PAGAL) ^14^, thus identifying potential targets for inhibition of the helix mediated interactions. Through several examples, we demonstrate that helices with unique features are involved in interactions with host proteins (either antibodies from survivors, or proteins regulating the immune response). Further, we also provide an alternate way of analyzing differences in related proteins (from the Marburg virus) by focusing on the properties of corresponding helices. As future work, we intend to develop methodologies to design peptides that would target these ‘hotspots’ ^44^. It has to be kept in mind that it has been a challenge to design small ligands that disrupt protein-protein interactions, and designers resort to several innovative techniques to overcome thermodynamic instability or proteolytic susceptibility ^47– 50^. These helices can essentially be epitopes ^51, 52^ for developing antibodies against the virus ^53, 54^. Interestingly, ZMapp, a cocktail of three antibodies has shown reversion of advanced Ebola symptoms in non-human primates ^55^, and uses only glycoprotein-specific epitope generated antibodies ^52, 56^. It is interesting to hypothesize that additions to this cocktail with antibodies derived from other epitopes (for example, 4U2XA.HELIX5 from VP24 that is involved in immune response suppression) could prove more effective. Thus, we provide a comprehensive list of potential targets within the small proteome of the Ebola virus that can directed rational design to quickly innovate therapies.
