# Supplementary material for: Characterizing alpha helical properties of Ebola viral proteins as potential targets for inhibition of alpha-helix mediated protein-protein interactions
Source: F1000Res. 2015 Jan 27;3:251. Originally published 2014 Oct 24. [Version 3] doi: 10.12688/f1000research.5573.3 (PMC4329671; doi:10.12688/f1000research.5573.3)
Supplement: Peer review history

# Peer review: Referee response for version 3

**DOI:** 10.5256/f1000research.6510.r7576

**Published:** 2015-02-10

**Authors:** Sean Ekins

**Affiliations:** 1 Collaborative Drug Discovery, Burlingame, CA, USA

This publication is scientifically sound and has potential utility for those interested in identifying possible target binding sites and for molecular modification studies e.g. site directed mutagenesis.

It would also have been useful to test other computational methods besides PAGAL or even describe the pros/cons of these. Could they be used in consensus to improve predictions? Perhaps the authors can comment on this.

I would clarify that our docking and pharmacophore computations (Ekins *et al *ref) only suggests FDA drugs chloroquine/ amodiaquine  may bind VP35. It is clear from the x-ray structures in the PDB that several drug-like molecules can bind. We also have yet to verify our predictions but thank you for citing this.

This study does make you wonder too what else could be done computationally for Ebola research, what should be most helpful for drug discovery?

Probably a logical step might be to identify potential binding locations on the alpha helices for small molecules and possibly dock compounds at these sites.

The authors clearly describe their approach and present results which can be tested by them or others in future. With limited published scientific knowledge on the Ebola virus it is important that such computational approaches are used.

I have read this submission. I believe that I have an appropriate level of expertise to confirm that it is of an acceptable scientific standard.

# Peer review: Referee response for version 2

**DOI:** 10.5256/f1000research.6147.r7109

**Published:** 2014-12-19

**Authors:** Winfried Weissenhorn

**Affiliations:** 1 Unit for Virus Host-Cell Interactions (UVHCI), Grenoble, France

The authors have responded to my previous questions and improved the manuscript. Although, the described computational approach can identify alpha helices with unique features, the author’s proposal that the helical propensities can be linked to host protein interactions is rather weak and requires experimental data to validate the method.

There are still a conceptual error in the manuscript:

Page 4: " *The antibody most likely inhibits the rearrangement of GP2 segments, which abrogates the fusion of the internal loop in the host membrane*."

Do the authors mean that the antibody binding prevents Gp2 conformational changes required for membrane fusion?

I have read this submission. I believe that I have an appropriate level of expertise to confirm that it is of an acceptable scientific standard.

# Response

**Published:** 2015-01-26

**Authors:** Sandeep Chakraborty

**Affiliations:** Tata Institute of Fundamental Research, India

Dear Dr Weissenhorn,

    We appreciate your positive comments to the changes we had made in accordance to your previous suggestions.

By the statement `The antibody most likely inhibits the rearrangement of GP2 segments, which abrogates the fusion of the internal loop in the host membrane.', we meant the same thing as `antibody binding prevents Gp2 conformational changes required for membrane fusion', which is the accepted hypothesis. We could not detect any difference in the meaning of the two wordings.

Please also find a new version of the manuscript which includes further corroboration of our hypothesis.

best regards,

Sandeep

# Peer review: Referee response for version 1

**DOI:** 10.5256/f1000research.5956.r6506

**Published:** 2014-11-04

**Authors:** Winfried Weissenhorn

**Affiliations:** 1 Unit for Virus Host-Cell Interactions (UVHCI), Grenoble, France

The authors suggest that they can identify alpha helices and predict their propensities to be targeted by small molecules. Their test case is the small Ebola virus genome, where several crystal structures are available.

First they compute the hydrophobic moment of identified helices with their previously published program PAGAL and classify them based on hydrophobicity, positive or negative charges. They conclude that helices with unique feature values are involved in host protein interaction.

Page 4: It is not correct to state that “ this helix is disrupted by a neutralizing antibody derived from a human survivor …”. HR1 or helix 1 from Gp2 is split into 4 small helices in the native GP structure and antibody binding prevents its refolding into the post fusion conformation represented by the Gp2 structure. Now one can argue that small molecules could interfere with the formation of the triple stranded coiled coil formed by HR1 in the post fusion structure. This needs to be clarified in the text.

Next they identified a charged helix in Vps24 that interacts with karyopherin. Why was this chosen? Because of the available structure? This helix contains only two charged residues and would not fall under the classification of carrying a high charge!

The third helices described in detail are from Vps35 and the authors identify several helices with carry charges, but no clear targets are discussed.

Page 6: The authors make a connection between the number of acidic residues in a helix from Ebola Vps35 compared to Marburg Vps35 and the frequency of outbreaks, which is a complete over interpretation of their data.

In summary the manuscript describes an interesting approach to identify or validate potential drug targets. However, the authors need to be more cautious in interpreting their results. Without any experimental validation their approach to link helical properties to protein interaction propensities is extremely weak.

I have read this submission. I believe that I have an appropriate level of expertise to confirm that it is of an acceptable scientific standard, however I have significant reservations, as outlined above.

# Response

**Published:** 2014-11-06

**Authors:** Sandeep Chakraborty

**Affiliations:** Tata Institute of Fundamental Research, India

Dear Dr Weissenhorn,

We would like to thank you for taking the time to review this paper, and for your suggestions to improve the manuscript. In the interim period, we have applied other computational methods[ref-1] to correlate the different immunosuppressive and pathogenicity mechanisms in Ebola and Marburg viruses to variations in their structures/sequences [ref-2]. Please find our detailed responses to your

comments below.

**The authors suggest that they can identify alpha helices and predict their propensities to be targeted by small molecules. Their test case is the small Ebola virus genome, where several crystal structures are available.**

**First they compute the hydrophobic moment of identified helices with their previously published program PAGAL and classify them based on hydrophobicity, positive or negative charges. They conclude that helices with unique feature values are involved in host protein interaction.**

**Page 4: It is not correct to state that this helix is disrupted by a neutralizing anti- body derived from a human survivor . HR1 or helix 1 from Gp2 is split into 4 small helices in the native GP structure and antibody binding prevents its refolding into the post fusion conformation represented by the Gp2 structure. Now one can argue that small molecules could interfere with the formation of the triple stranded coiled coil formed by HR1 in the post fusion structure. This needs to be clarified in the text.**

We appreciate this point, (‘ *KZ52 likely neutralizes by preventing rearrangement of the GP2 HR1A/HR1B segments and blocking host membrane insertion of the internal fusion loop*’ [ref-3]), and have made the correction.

**Next they identified a charged helix in Vps24 that interacts with karyopherin. Why was this chosen? Because of the available structure? This helix contains only two charged residues and would not fall under the classification of carrying a high charge!**

VP24 came up in the sorted list since it has a ‘high proportion of negatively charged residues’, and not high charge. The proportion of charged residues is computed based on the total number of charged residues, and not the length of the helix. We could also create a category of high charge by combining the previous feature (high proportion) to high number of charged residues.

Our search criteria excludes AHs with zero or one charged residue. We had stated this in the Methods section - We ignore the helices that have none or a single charged residue. We also had a cutoff on the length of the AH as 10 - i.e. we are looking for reasonably long AHs - we had not mentioned this constraint. We have modified the Methods section to reflect this. An AH having just two similarly charged residues in a reasonably long AH (and none other) is relatively significant. For example, one charged residue in VP24 (D124) makes an electrostatic contact with human karyopherin, while the other one E113 makes a contact to Arg140 in another helix (α6) in VP24 [ref-2].

**The third helices described in detail are from Vps35 and the authors identify several helices with carry charges, but no clear targets are discussed.**

We have stated that ‘we have not been able to identify a critical role for this helix in the protein from current literature’, which does not preclude the importance of these helices. This, in fact, highlights the ability of our method to extract helices that might be of significance, yet not probed sufficiently as targets. At the same time, it is also equally possible that this helix is not functionally significant.

**Page 6: The authors make a connection between the number of acidic residues in ahelix from Ebola Vps35 compared to Marburg Vps35 and the frequency of outbreaks, which is a complete over interpretation of their data.**

We agree with this criticism, and have made the corrections.

**In summary the manuscript describes an interesting approach to identify or validate potential drug targets.**

We appreciate the positive and encouraging note on our efforts to use computational methods to identify critical regions of interaction in the Ebola proteins, which could be easily extended to other organisms as well.

**However, the authors need to be more cautious in interpreting their results. Without any experimental validation their approach to link helical properties to protein interaction propensities is extremely weak.**

We hope that we have addressed your concerns by the changes that we have made. We also expect future results to corroborate some of our predictions, and will make the updates on the f1000 site (which their format allows us to). We sincerely hope that the manuscript will be found suitable in the modified form for publication.

Thanking you,

Sincerely,

Sandeep Chakraborty (Corresponding author)

[References]

[[1|type=journal|title=DOCLASP - Docking ligands to target proteins using spatial and electrostatic congruence extracted from a known holoenzyme and applying simple geometrical transformations [v1; ref status: awaiting peer review, http://f1000r.es/48g]|authors=Chakraborty/S|source=F1000Research|year=2014|vol=3|issue=262|doi=10.12688/f1000research.5145.1|url=http://f1000research.com/articles/3-262/v1]]

[[2|type=journal|title=Correlating the ability of VP24 protein from Ebola and Marburg viruses to bind human karyopherin to their immune suppression mechanism and pathogenicity using computational methods [v1; ref status: awaiting peer review, http://f1000r.es/4o3]|authors=Chakraborty/S|source=F1000Research|year=2014|vol=3|issue=265|doi=10.12688/f1000research.5666.1|url=http://f1000research.com/articles/3-265/v1]]

[[3|type=journal|title=Structure of the Ebola virus glycoprotein bound to an antibody from a human survivor|authors=Lee/JE;Fusco/ML;Hessell/AJ;Oswald/EB;Burton/DR;Saphire/EO|source=Nature|year=2008|vol=454|issue=7201|fpage=177|lpage=182|pmid=18615077|pmcid=2700032|doi=10.1038/nature07082|url=http://www.nature.com/nature/journal/v454/n7201/full/nature07082.html]]
